# Experimental Evaluation of 5G NR OFDM-Based Passive Radar Exploiting Reference, Control, and User Data

**DOI:** 10.3390/s26041317

**Published:** 2026-02-18

**Authors:** Marek Wypich, Tomasz P. Zielinski

**Affiliations:** 1 Faculty of Computer Science, Electronics and Telecommunications, Institute of Telecommunications, AGH University of Krakow, al. A. Mickiewicza 30, 30-059 Krakow, Poland; marekwypich@agh.edu.pl; 2 Ericsson, ul. Konstruktorska 12, 02-673 Warszawa, Poland

**Keywords:** integrated sensing and communication (ISAC), 5G New Radio (NR), passive radar, OFDM radar, illumination of opportunity, reference signals, user data payloads, delay-Doppler map (DDM), channel frequency response (CFR), bit error rate (BER)

## Abstract

In communication-centric integrated sensing and communication (ISAC) systems, passive radars exploit existing communication signals of opportunity for sensing. To compute delay-Doppler or range–velocity maps (DDMs and RVMs, respectively), modern orthogonal frequency division multiplexing (OFDM)-based sensing systems use the channel frequency response (CFR) originally estimated in communication receivers for equalization. In OFDM-based passive radars utilizing 4G LTE or 5G NR waveforms, CFR estimation typically relies only on reference signals. However, simulation-based studies that assume a priori knowledge of user data symbols indicate potential performance gains when incorporating user data and other downlink channels. In this work, we present an experimental evaluation of an OFDM-based passive radar that jointly utilizes all commonly present components of the 5G NR downlink waveform: synchronization signals (PSS and SSS), broadcast and control channels (PBCHs and PDCCHs, respectively), data channels (PDSCHs), and reference signals (PBCH DM-RSs, PDCCH DM-RSs, PDSCH DM-RSs, and CSI-RSs). Our results show that utilizing user data from fully occupied 5G downlink signals, under the assumption of full knowledge of PDSCH locations, significantly improves both the probability of detection (POD) and the peak height, measured by the peak-to-noise-floor ratio (PNFR), compared with pilot-only sensing. Since perfect knowledge of the user data payload is not assumed, we estimate the transmission bit error rate (BER) and analyze its impact on sensing performance. Finally, we investigate more realistic scenarios in which only a subset of PDSCH resource element locations is known, as in practical 5G deployments, and evaluate how partial data location knowledge affects the POD and PNFR under different BER conditions.

## 1. Introduction

The concept of using a single waveform for both radar and communication functions has been extensively studied for many years, with numerous key milestones and a rich research history [1,2]. Three primary designs have been identified in this domain: radar-centric, communication-centric, and joint designs [3,4]. Given the broad scope of research in this area, our focus is limited to the communication-centric approach. In communications, integrated sensing and communication (ISAC) has attracted significant attention, as it is envisioned to be one of the key enablers of 6G networks [3,4,5]. Moreover, it has already been incorporated as a study item [6] within the 3GPP standardization efforts for 5G, underscoring its growing importance in future wireless systems.

Numerous studies have explored various approaches to utilizing 5G signals as illuminators of opportunity for radar systems. The authors of [7] discussed several applications, including active sensing, where the base station transmits and receives radar pulses, and passive sensing, where an independent ISAC receiver listens to the 5G downlink and uses it for target detection. In passive detection [8], the predominant technique is based on the cross-ambiguity function (CAF), which relies on correlating the received 5G downlink signal reflected from potential targets with the line-of-sight (LOS) signal recorded by a separate antenna (e.g., [9]).

An alternative approach uses the fact that in modern wireless communication systems, such as Digital Audio Broadcasting (DAB), Digital Video Broadcasting-Terrestrial (DVB-T/T2), Wi-Fi, and 4G or 5G, orthogonal frequency division multiplexing (OFDM) [10] is the dominant transmission technology. In OFDM systems, in addition to user and control channels, the base station transmits reference signals, namely sequences known to the receiver. In receivers, they are used to estimate the channel frequency response (CFR) and to compensate for channel effects during data demodulation. This waveform design enables an OFDM-based sensing technique [11,12], in which the CFR is further analyzed to extract information about moving objects in the vicinity of the receiver.

Several studies have demonstrated sensing results for OFDM-based radars utilizing 5G downlink broadcast, control, and reference signals, including the primary and secondary synchronization signals (PSSs and SSSs, respectively), physical broadcast channel (PBCH), physical downlink control channel (PDCCH), channel state information reference signals (CSI-RSs), demodulation reference signals (DM-RSs), phase tracking reference signals (PT-RSs), tracking reference signals (TRSs), and positioning reference signals (PRSs). In [13], the ranging error of the PSS, PBCH DM-RS, and PRS was analyzed in ray-tracing simulations. The authors of [14] showed the superiority of the 5G PRS, compared with a PSS, SSS, DM-RS and CSI-RS, in range and velocity estimation. In [15], different PRS configurations were further examined for their impact on the root mean square error (RMSE) and peak-to-noise-floor ratio (PNFR) in velocity estimation. Khosroshahi et al. [16] proposed a joint PRS and PDSCH DM-RS utilization method that further enhances the sensing capabilities. In addition, the authors of [17] discussed the characteristics of various 5G pilots (CSI-RS, DM-RS, PT-RS, TRS, and PRS), analyzed in simulations the joint usage of DM-RSs and CSI-RSs as the most promising option for radar due to their regularity and density, and presented experimental results demonstrating successful vehicle detection. Moreover, in our previous publications [18,19], we demonstrated that 5G signals, including CSI-RS and custom radar-on-demand (RoD) pilots, can be used to detect moving vehicles, both in simulations and in real-world field measurements.

Although reference signals are promising candidates for the introduction of sensing capabilities into existing communication infrastructures, they are typically sparsely distributed within the OFDM grid. This sparsity introduces several limitations, such as a reduced maximum measurable velocity and a lower signal energy available for target illumination. Since communication networks are primarily designed to transmit user data, these data payloads occupy most of the OFDM resources. This poses a significant challenge, as unlike pilots, which are pseudo-random sequences with well-defined correlation properties, user data consist of random sequences that are unknown in passive radars. Additionally, repurposing data payloads and control channels for sensing can result in non-uniform sampling of the CFR.

To address these challenges, several studies have investigated the use of user symbols for radar sensing, which in 5G systems are carried out in the physical downlink shared channel (PDSCH). The authors of [20] provided a technical overview of sensing and communication (S & C) using both reference signals and user data. In [21], sensing with 4G LTE and 5G NR waveforms was analyzed; however, the relative contribution of individual downlink components to radar performance was not distinguished. In [22,23], simulation results showing that repurposing user data for radar when the sensing device has a priori knowledge of the transmitted bits significantly enhances radar performance are presented. The authors of [24] demonstrated how the joint use of DM-RSs, CSI-RSs, and data payloads can further improve sensing capabilities. The impact of reconstruction errors was examined in [25] for WiFi-like signals; however, this work relied on the assumption that user data occupy all non-pilot resources. In [26], an iterative OFDM-based sensing approach was proposed for multi-target scenarios that takes advantage of both pilots and user data. Furthermore, in our previous work [27], we showed in simulations that repurposing user data OFDM resources for radar applications can enhance sensing performance. Through simulations, we examined the impact of different QAM modulation orders of data payloads and analyzed how transmission errors, measured by the bit error rate (BER), affect radar sensing capabilities.

The aim of this paper is to experimentally evaluate the potential of joint exploitation of user data payloads and other commonly present 5G downlink waveform components for sensing purposes. In our field measurements, we performed successful transmission and communication processing using three different 5G signals containing synchronization and broadcast information (PSS, SSS, and PBCH), control information (PDCCH), pilot signals (CSI-RS and DM-RS), and user data (PDSCH). We then performed OFDM-based sensing using various combinations of these components and compared the results. Our primary focus is on user data, as they occupy most of the 5G OFDM resources and can provide a significant performance boost over pilot-only approaches. We exclude PRSs, PT-RSs, and TRSs from the scope of this study. Although some works report performance gains when sensing with these signals, they are rarely transmitted by base stations, which typically minimize non-data signaling to maximize throughput.

A key contribution of this work is that the analysis is based on real-world field measurements. Most existing studies [13,14,15,16,18,22,23,25,26] investigate this concept primarily through simulations, with only a few [17,19,21,24] including experimental validation. Field measurements introduce additional challenges, such as hardware impairments, synchronization errors, and channel dynamics, which are typically omitted in simulation-based analyses. The novelty of this work with respect to the prior art can be summarized as follows:

1. **Field measurements with joint exploitation of multiple 5G downlink components:** While prior studies have investigated sensing with individual 5G reference signals [14,15,18,19] or joint pilot-and-data approaches [13,16,17,24], these works are limited to specific signal subsets. Unlike [21], which presents a general waveform analysis, our present work provides a comprehensive field evaluation of various combinations of PSSs, SSSs, PBCHs, PDCCHs, PDSCHs, DM-RSs, and CSI-RSs. Our measurements demonstrate that incorporating reconstructed user data improves the probability of detection (POD) from approximately 24–32% (using only pilots or a control) to 77–78% (using all components) and reduces the mean PNFR deviation relative to the ground truth from about 6.5 dB to 2.5 dB under the assumption of a fully loaded waveform with perfect knowledge of signal component locations.
2. **Experimental BER-to-sensing degradation analysis:** The existing literature often assumes a priori knowledge of user payloads [22,23] or error-free transmission [24]. While the authors of [25] explored symbol error rates for WiFi-like signals in simulations, and the authors of [26] proposed iterative error mitigation, the present work explicitly measures the impact of real-world transmission errors on 5G-based radar performance. We establish experimentally that sensing remains robust (<0.5 dB PNFR loss) at low bit error rates ($BER<10^{-2}$), while higher error rates ($BER>10^{-1}$) result in a performance degradation of approximately 4.5–5 dB compared with the genie-aided baseline.
3. **Analysis of sensing performance under partial knowledge of PDSCH resource locations:** Common models in the current literature, such as [25], typically assume that user data occupy all non-pilot resources. This is often unrealistic for passive receivers in multi-user or low-load networks. We address this gap by evaluating sensing performance when only a subset of PDSCH locations is known. Our analysis reveals that knowledge of just 20% of PDSCH resources (under low-BER conditions) or 40% (under high-BER conditions) is sufficient to achieve a POD exceeding 96%. Although these absolute values reflect specific measurement conditions, they illustrate the general potential of effective sensing using only a fraction of decoded user traffic.

The remainder of this paper is organized as follows. Section 2 presents the mathematical foundation of OFDM-based sensing in a fading channel. Section 3 describes the 5G ISAC receiver architecture, including the communication subsystem (Section 3.1) and the OFDM-based sensing module (Section 3.2). In Section 4, we present the experimental sensing results obtained from our field measurements with different 5G signals. The geometry of the experiment is described in Section 4.1, different radar configurations are discussed in Section 4.2, the (genie-aided) ground-truth calculation method is presented in Section 4.3, radar performance analysis is provided in Section 4.4, the impact of the BER on radar performance is examined in Section 4.5, and radar accuracy in scenarios with different numbers of known user data locations is analyzed in Section 4.6. We also present the initial simulation results showing radar performance for different PDSCH QAM orders in Section 5. Finally, Section 6 provides the conclusions, summarizing our findings and outlining potential directions for future research.

## 2. OFDM-Based Sensing Principle

The received signal y(t), resulting from the transmission of x(t) through a linear time-variant (LTV) radio channel [10,28], can be modeled as a sum of *P* reflected paths, indexed by p=1,…,P, and an LOS path indexed by p=0:

(1) $$y\left(t\right)=\sum_{p=0}^{P}g_{p}x\left(t-\tau_{p}\right)e^{j2\pi f_{p}^{\left(D\right)}t}.$$

Each path is characterized by a complex attenuation coefficient $g_{p}$, a delay of $\tau_{p}$ seconds, and a Doppler frequency shift $f_{p}^{\left(D\right)}$ in Hz. For the LOS path and reflections from static objects, the Doppler shift $f_{p}=0$. Equation (1) can be rewritten in its integral form as

(2) $$y\left(t\right)=\int_{-\infty }^{\infty }h\left(t,\tau \right)\;x\left(t-\tau \right)\;d\tau ,\;$$

which generalizes the linear time-invariant (LTI) case, where h(t,τ) reduces to h(τ). Equation (2) describes the received signal as the convolution of the channel impulse response (CIR) with the transmitted signal. The time-variant (TV) CIR can then be expressed as

(3) $$h\left(t,\tau \right)=\sum_{p=0}^{P}g_{p}\;e^{j2\pi f_{p}^{\left(D\right)}t}\;\delta \left(\tau -\tau_{p}\right).\;$$

Since paths resulting from reflections from moving objects exhibit non-zero Doppler shifts, the complex gain of each path oscillates as a function of time. These oscillations appear in the TV-CIR at each delay tap $\tau =\tau_{p}$. To extract the Doppler shift frequency $f_{p}^{\left(D\right)}$ for the *p*th path, the TV-CIR can be analyzed along the time dimension *t* using a Fourier transform. The resulting Doppler shifts can then be converted to velocities of the reflecting objects, while the time delays $\tau_{p}$ provide estimates of the total propagation path lengths.

**Figure 1:**
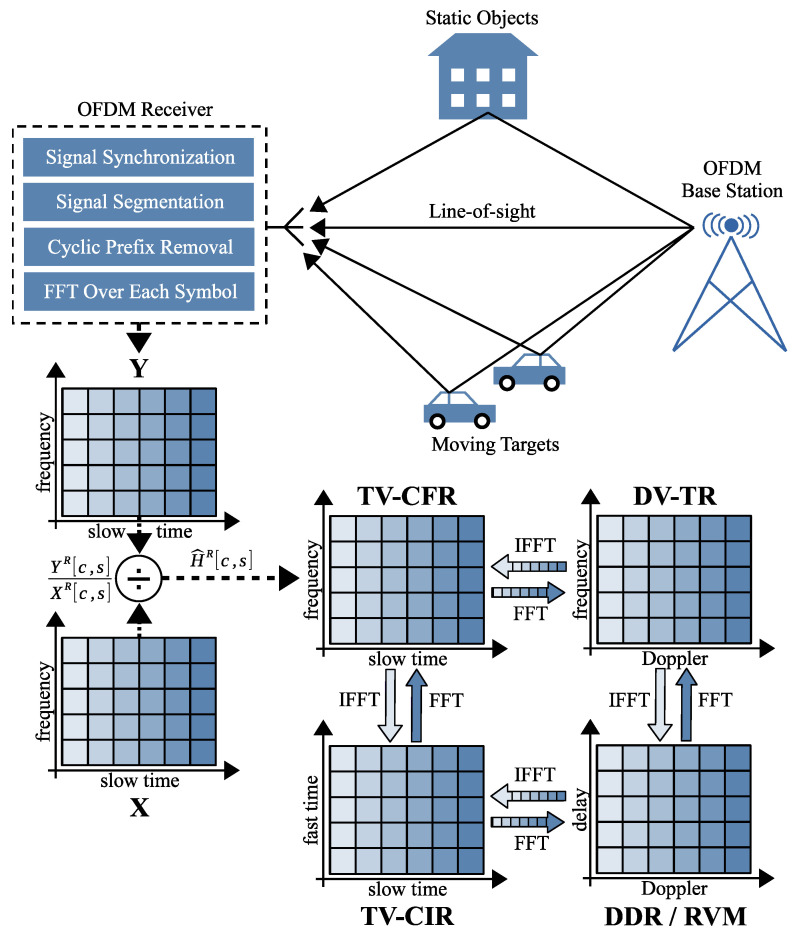
OFDM-based sensing. Matrix Y of the received carrier states is decoded based on real measurements, while matrix X of the transmitted carrier states is known to the receiver. The RVM is computed by performing an IFFT over TV-CFR matrix columns and an FFT over TV-CIR matrix rows.

In OFDM systems, the sampled received signal y[n] is processed as follows (see Figure 1). First, the stream is divided into segments corresponding to individual OFDM symbols including a cyclic prefix (CP). After cyclic prefix removal, each symbol of a length *N* (where *N* is the FFT size) is shaped into a two-dimensional matrix y[n,s], where the first dimension represents time-domain samples within a symbol, and the second dimension represents the symbol index. Each symbol then undergoes a fast Fourier transform (FFT) to produce the OFDM resource grid Y[c,s] in the frequency domain, where *c* denotes the subcarrier index and *s* denotes the OFDM symbol index. This process is explained in more detail using the 5G waveform example in Section 3.1. Channel estimation is essential for equalizing user data. For this purpose, known pilot sequences are embedded at predetermined positions *R* (Reference) in the transmitted grid X[c,s]. The CFR at pilot locations is estimated using

(4) $${\hat{H}}^{R}\left[c,s\right]=\frac{Y^{R}\left[c,s\right]}{X^{R}\left[c,s\right]},\;\left(c,s\right)\in R.\;$$

The full TV-CFR estimate $\hat{H}\left[c,s\right]$ is then obtained by applying two-dimensional interpolation to ${\hat{H}}^{R}\left[c,s\right]$.

Since the TV-CFR is computed in every modern OFDM-based communication system, it becomes straightforward to integrate radar functionality using the same CFR samples, as illustrated in the lower part of Figure 1. First, the TV-CFR is obtained by dividing the received carrier states $Y^{R}\left[c,s\right]$ by the known transmitted reference values $X^{R}\left[c,s\right]$. The TV-CIR is then generated by applying an IFFT to each column of the CFR matrix. Finally, each row of the resulting CIR matrix is analyzed using an FFT to extract the frequencies of potential oscillations, producing the delay–Doppler response (DDR). The DDR axes can be converted to range and velocity, forming a range–velocity map (RVM). The RVM peaks indicate the distance (in meters) and speed (in meters per second) of moving objects near the receiver. It is also important to note that an equivalent result can be obtained by following an alternative processing path based on the Doppler-variant transfer response (DV-TR).

## 3. 5G NR ISAC Receiver

### 3.1. Communication

**Figure 2:**
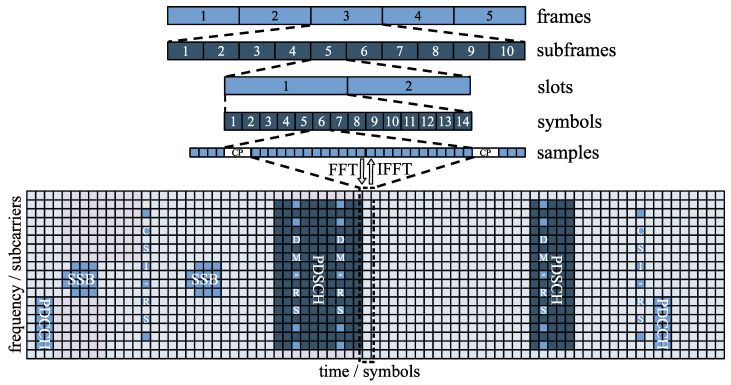
5G NR resource time–frequency (OFDM symbols-carriers) grid for SCS = 30 kHz. From top to bottom: one frame consists of 10 subframes, one subframe contains 2 slots, one slot comprises 14 OFDM symbols, and each symbol spans $N_{\mathrm{fft}}$ subcarriers. Time-domain OFDM symbols are transformed via FFT to obtain the time–frequency grid. In the grid representation, gray indicates unused (empty) resource elements, while blue denotes active components of the 5G NR waveform, including SSB, PDCCH, PDSCH, CSI-RS, and DM-RS.

Figure 2 presents an example of the OFDM resource grid of a 5G waveform. The 5G NR signal is structured into 10 ms frames, each divided into 10 subframes of 1 ms duration ([4.3.1 Frames and subframes] of Ref. [29]). Each subframe is further divided into slots, determined by the numerology parameter μ ([Section 4.3.2. Slots] in [29]). In this work, we set μ=1, which corresponds to a subcarrier spacing (SCS) of 30 kHz and results in two 0.5 ms slots per subframe ([Section 4.2. Numerologies] in [29]). Each slot consists of 14 OFDM symbols.

The 5G OFDM resources can be represented on a two-dimensional grid, where the *x* axis corresponds to time (consecutive OFDM symbols) and the *y* axis corresponds to the frequency (OFDM carriers). Each point in this grid is called a resource element (RE) ([Section 4.4.3. Resource elements] in [29]) and is typically modulated using various orders of quadrature amplitude modulation (QAM) ([Section 5.1. Modulation mapper] in [29]) to convey information. Before transmission, each symbol (i.e., each column of the grid) undergoes an IFFT. Then, a portion of the end of the resulting time-domain vector is copied to its beginning to form a cyclic prefix, which helps mitigate intersymbol interference (ISI). In 5G, the CP of the first symbol in certain slots is longer than that of the remaining symbols ([Section 5.3. OFDM baseband signal generation] in [29]). Finally, all modulated symbols are concatenated to construct the complete time-domain signal.

**Figure 3:**
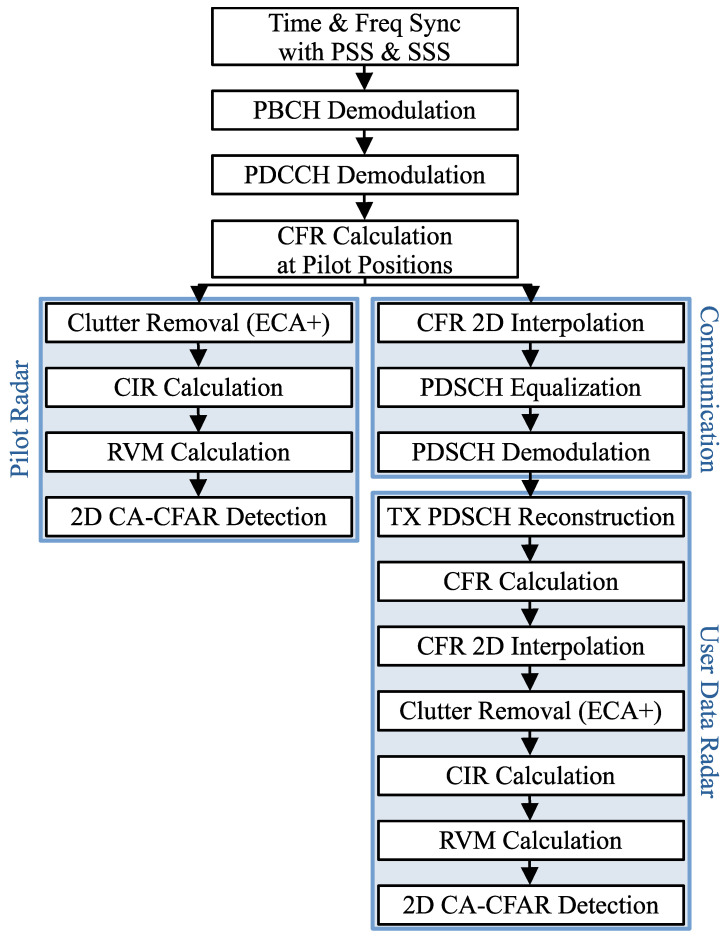
Operations performed in the 5G NR-based ISAC receiver.

**Figure 4:**
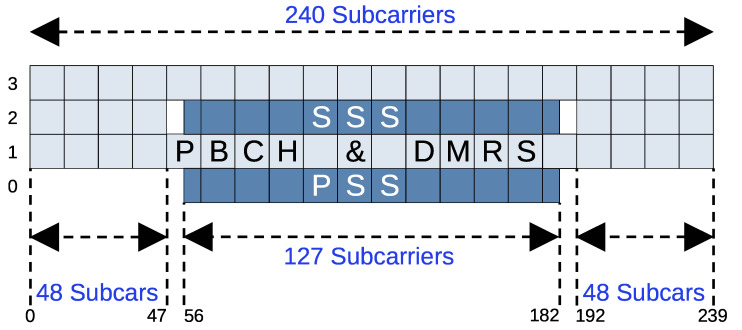
Structure of the 5G synchronization signal block (SSB).

To successfully decode data, a 5G receiver must perform several steps to reverse the transmitter’s modulation process. A simplified block diagram of these steps is shown in Figure 3. The first task is time and frequency synchronization, which is accomplished using the PSS. The PSS is located in the first symbol (index 0) of the synchronization signal block (SSB) ([Section 7.4.3.1. Time-frequency structure of an SS/PBCH block] in [29]), which is presented in Figure 4. SSBs are built from four symbols that occupy 240 subcarriers and are typically transmitted in bursts with a size of one to $L_{max}$. These bursts are periodically repeated, with a commonly used repetition period of 20 ms ([Section 4.1. Cell search] in [30]). Although their timing positions within this period are fixed, their frequency locations can vary depending on the Global Synchronization Channel Number (GSCN) raster ([Section 5.4.3. Synchronization raster] in [31]). To achieve synchronization, the receiver correlates the three known PSS sequences with the received signal to determine the SSB’s frequency position, the coarse (integer) carrier frequency offset (CFO), and its time position within the received frame. The synchronization procedure is then refined by performing a more precise fine (fractional) CFO estimation using CPs [32].

The next step is the demodulation of the PBCH. However, before this can be performed, the Cell ID must first be determined. This is accomplished using the SSS, which is located in the third symbol (index 2) of the SSB ([Section 7.4.3.1. Time-frequency structure of an SS/PBCH block] in [29]). The SSS transmits 1 of 336 binary m-sequences ([Section 7.4.2.3. Secondary synchronization signal] in [29]). By performing a blind search, i.e., correlating the known SSS sequences with the received one, the receiver identifies the $N_{ID}^{\left(1\right)}$ value. Similarly, the $N_{ID}^{\left(2\right)}$ value can be determined using the PSS, which transmits one of three different m-sequences ([Section 7.4.2.2. Primary synchronization signal] in [29]). The complete Cell ID is then computed as defined in Section 7.4.2.1. Physical-layer cell identities in [29]):

(5) $$N_{CellID}=3\times N_{ID}^{\left(1\right)}+N_{ID}^{\left(2\right)}.$$

The remaining REs of the SSB are occupied by the PBCH and its associated DM-RS ([Section 7.4.3.1. Time-frequency structure of an SS/PBCH block] in [29]). After PBCH demodulation and broadcast channel (BCH) decoding ([Section 7.1. Broadcast channel] in [33]), the Master Information Block (MIB) can be extracted to obtain essential cell configuration information ([Section 6.2.2. Message definitions] in [34]). Notably, the entire SSB is the only always-on signal in the 5G NR system.

Once synchronized and equipped with the initial system information, the 5G receiver proceeds to acquire additional details regarding the exact locations of the transmitted user data. This information is transported through the PDCCH ([Section 7.3.2. Physical downlink control channel (PDCCH)] in [29]). Since the position of the PDCCH within the resource grid is not fixed, the receiver must perform blind detection across predefined PDCCH search spaces, which specify potential regions where the PDCCH may be located ([Section 10.1. UE procedure for determining physical downlink control channel assignment] in [30]). The PDCCH carries downlink control information (DCI), which contains instructions about the physical resource allocation of user data ([Section 7.3. Downlink control information] in [33]). To successfully decode the DCI, the receiver must know the Radio Network Temporary Identifier (RNTI), since the cyclic redundancy check (CRC) of each DCI message is scrambled using this number ([Section 7.3.2. CRC attachment] in [33]). There are several types of RNTIs ([Section 7.1. RNTI values] in [35]); some are common RNTIs known to all users and used to scramble DCIs containing broadcast or system-wide information, and others are user-specific RNTIs, which are known only to individual users, allowing them to decode their respective DCIs and identify the locations of their assigned data ([Section 5.1. Random Access procedure] in [35]). Finally, once the DCI has been successfully decoded, the receiver obtains the necessary information to access the user data, which is transmitted through the PDSCH ([Section 7.3.1. Physical downlink shared channel] in [29]).

Unlike 4G LTE, where cell-specific reference signals (CRSs) were continuously transmitted at fixed positions across the entire OFDM grid, 5G does not include any always-on reference signals for channel estimation. Instead, each physical channel defines its own set of DM-RSs, which are transmitted among other symbols, such as PBCH DM-RSs, PDCCH DM-RSs, and PDSCH DM-RSs ([Section 7.4.1. Reference signals] in [29]). Before demodulating the channel symbols, the receiver estimates the CFR using these pilots. Then it is interpolated over the remaining REs and used for equalization, i.e., to mitigate the impact of the channel on the received data. Another type of reference signal present in the 5G waveform is the CSI-RS ([Section 7.4.1.5. CSI reference signals] in [29]). It is transmitted by the gNodeB (5G base station) to enable estimation of the downlink radio channel quality. Again, the key difference from the LTE CRS is that the 5G CSI-RS is configured per device rather than per cell, which means it is not continuously transmitted and may not always be present in the signal.

### 3.2. OFDM-Based Sensing

The CFR estimates, which are already computed in every 5G communication device, can be reused for sensing applications. This concept is illustrated in Figure 3, where after the initial three processing steps, the diagram branches into two directions: one for standard communication processing and the other for radar. In the sensing branch, the first stage is clutter removal. Clutter appears as strong peaks near the zero-Doppler region, originating from the LOS path, and reflections from static objects such as buildings or trees. Since clutter often has significantly higher energy than weaker target reflections, it can mask moving targets and must therefore be removed from the RVM. In our work, we tried several variants of the Extensive Cancellation Algorithm (ECA) [36], specifically ECA by Carrier and Doppler (ECA-CD) [37] and ECA+ [38,39]. Ultimately, we selected ECA+ because of its low computational complexity and strong clutter suppression performance. Once clutter is removed, additional OFDM-based radar processing is applied, as described in Section 2. In our previous studies [18,19], we demonstrated that this approach, using CFRs derived from 5G CSI-RSs and our custom RoD pilots, can effectively detect moving vehicles. Since the CFR in this method is computed using pilot signals, we refer to it as pilot radar (P-Radar) and the resulting RVM as pilot RVM (P-RVM).

During signal reception, the 5G receiver demodulates and decodes various parts of the waveform, as described in Section 3.1. This enables reconstruction of the transmitted symbols at those locations, allowing them to be reused as pilot-like symbols to calculate a denser CFR estimate for sensing purposes. This process is illustrated in the bottom-right section of Figure 3. However, unlike the pilot-only CFR, this reconstructed CFR is non-uniformly sampled, since the positions of all physical channels and signals, particularly the PDSCH, may appear random from a radar perspective. To address this, two-dimensional interpolation for non-uniformly distributed samples is applied to fill in the missing values. In this work, a linear interpolation method is used [40]. After interpolation, the standard OFDM-based sensing procedure is performed, including clutter removal and RVM generation. The resulting RVM, referred to as the user data RVM (UD-RVM), is constructed from a significantly larger number of samples than the P-RVM, resulting in improved sensing performance. In our previous work [27], we presented simulation results demonstrating that the use of UD-RVMs enhances detection accuracy and enables the detection of high-speed targets that cannot be captured using P-RVMs.

## 4. Field Measurements

**Figure 5:**
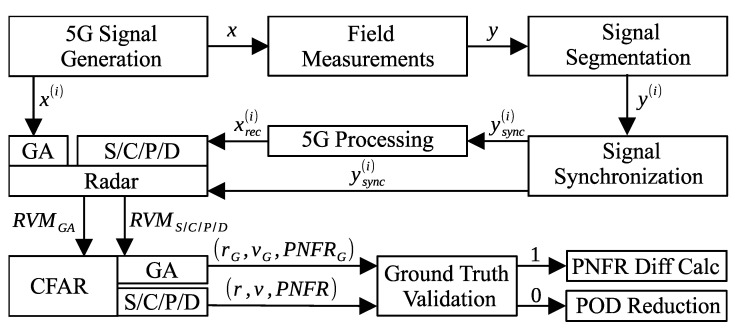
Experiment methodology. Each generated 5G waveform was transmitted over a real radio channel and recorded. The received stream was segmented and synchronized using the 5G SSB, after which a reconstructed transmit copy was obtained through 5G communication processing. Radar sensing was then performed using the reconstructed and received data. Genie-aided (GA) ground-truth values were obtained by replacing the reconstructed copy with the original error-free waveform. Detections from each radar configuration were validated against the GA reference (1—valid, 0—not valid), and PNFR and POD statistics were computed.

**Figure 6:**
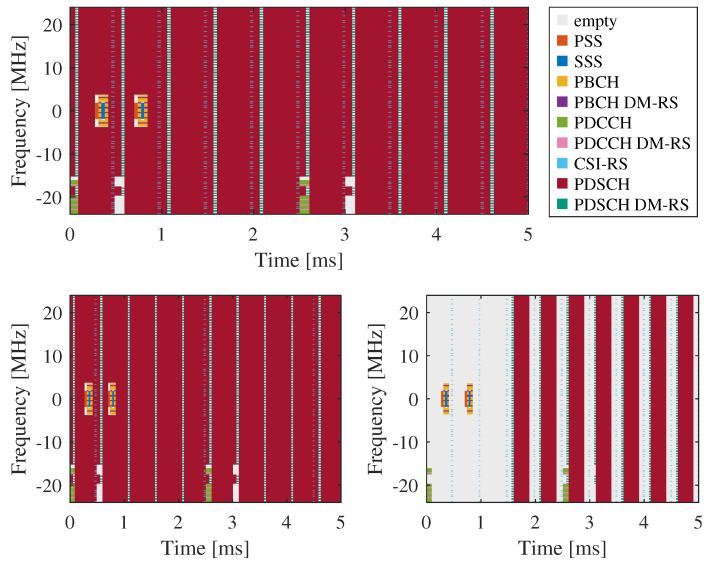
Time–frequency (symbol-carrier) grids of 5G signals used in field measurements: signal A (**top**), signal B (**bottom-left**), and signal C (**bottom-right**).

**Figure 7:**
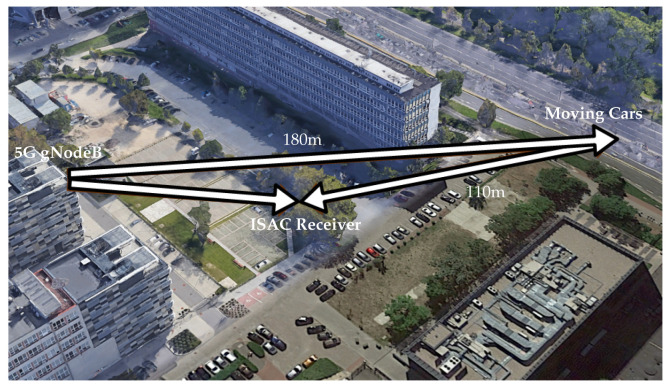
Set-up of the 5G NR-based passive bistatic radar experiment.

In this section, the results of the conducted field experiments are presented. The applied investigation methodology is presented in Figure 5. First, three different 5G NR downlink signals were generated using the MATLAB 2024b with 5G Toolbox version 24.2 (Figure 6, Table 1). These signals were then transmitted and recorded using two Software-Defined Radios (SDRs) arranged in a bistatic configuration (Figure 7, Table 2). This setup emulates a base station to user equipment (UE) transmission or a third-party ISAC receiver passively monitoring a 5G downlink signal. Each recorded signal was subsequently segmented into 10-frame intervals with a 5-frame step. For every segment, 5G communication processing was performed, including synchronization, demodulation, and decoding. This processing produced a reconstructed version of the transmitted signal, which may contain transmission errors. The BER was then computed by comparing the reconstructed signal with the originally transmitted one. Next, OFDM-based radar processing, including ECA+ clutter removal (Table 3) [38,39], followed by a constant false alarm rate (CFAR) algorithm (Table 4) [41], was applied to detect vehicles on a nearby high-speed road. Genie-aided ground-truth detections were established using a combination of the original transmitted signal and the received signal. All other radar configurations investigated in this study relied on the reconstructed (and potentially error-affected) signals together with the received signals for sensing. Finally, the detections produced by each radar configuration for all segments were validated using genie-aided ground-truth values. These values also served as a reference for performance evaluation, including the PNFR difference relative to the genie-aided radar and the POD, derived from the number of detections obtained with each tested radar.

### 4.1. 5G NR Configuration and Antenna Geometry

Our field measurements are intended to experimentally validate the proposed OFDM-based approaches that utilize different combinations of control, reference, and user data. During the experiments, we transmitted three different 5G signals generated using the MATLAB 5G Toolbox. The common components of these signals include two SSBs per SS burst transmitted every two frames and PDCCH transmissions occurring every 2.5 subframes. The signals differ in their CSI-RS and PDSCH configurations. All relevant 5G NR waveform generation parameters are summarized in Table 1, and the first 5 ms of each signal are illustrated in Figure 6.

The field measurement set-up was identical to that used in our previous work [19], and it is illustrated in Figure 7. The base station was emulated using an SDR positioned approximately 20 m above the ground. Before transmission, the signal was amplified using a high-power amplifier and transmitted through an antenna with a beamwidth of approximately 16° in both the horizontal and vertical planes and a directivity of approximately 19 dBi. We operated at a carrier frequency of 5.8 GHz, which lies within an unlicensed band. The ISAC receiver, located in the parking lot near the high-speed road, acquired the signal after it passed through a chain of low-noise amplifiers and bandpass filters between the antenna and the SDR, with a total amplifier gain of 20 dB. The SDR parameters are summarized in Table 2. The distance between the vehicles and the receiver was approximately 110 m, while the distance between the vehicles and the transmitter was about 180 m. Therefore, the total bistatic range [8] was estimated to be about 240 m.

Due to SDR limitations, the sampling rate used to transmit and record the signal (33.33 MHz) differed from that of the generated 5G signal (61.44 MHz). This resulted in improved velocity resolution due to longer symbol durations but a decreased range resolution and maximum detectable velocity, as summarized in Table 5.

### 4.2. Radar Configurations

Each signal captured by a receiver was segmented into 10 frame intervals with a 5 frame step. Each segment was first synchronized using the PSS, followed by cell identification using a combination of the PSS and SSS according to Equation (5). Subsequently, the PBCH was demodulated, and the MIB was decoded. To ensure correct signal reception, the BCH CRC was also verified. Since the main focus of this study was radar performance, subsequent communication stages were simplified. Instead of performing full PDCCH search space decoding and using DCI to locate the PDSCH, we utilized a priori knowledge of the PDCCH and PDSCH positions. However, unlike many studies that focus on the monostatic scenario, where the base station acts as both the transmitter and receiver or an idealized bistatic set-up with error-free knowledge of the transmitted data is assumed, we did not assume that the PDSCH symbol values were known at the receiver. Instead, we demodulated the received QAM symbols, reconstructed the transmitted signal through standard communication processing, and calculated the BER by comparing the reconstructed symbols with the ones originally generated.

This approach inherently assumes knowledge of all RNTIs present in the network, since such information is required for the receiver to identify all PDSCH locations. In practice, however, a UE connected to a 5G network is aware only of its own dedicated RNTI and a limited set of common system-wide identifiers, as discussed in Section 3.1. Moreover, when the network load is low, a large portion of the available resources may remain unused and not occupied by user data. Consequently, this assumption is valid only in a single-user scenario, where the ISAC receiver is the only user in a fully loaded network (an idealized and largely unrealistic case) or in offline sensing, where a blind search over all possible RNTIs may be feasible. Nevertheless, to demonstrate the potential benefits of exploiting user data for sensing and analyze the impact of the BER, we adopt this assumption in Section 4.4 and Section 4.5. However, in Section 4.6, we relax this constraint and evaluate radar performance when only a subset of PDSCH locations is known to the receiver, which more accurately reflects a realistic multi-user deployment.

In this study, we compare the sensing performance of OFDM-based radars that exploit different combinations of channels and signals available in the 5G NR downlink:

1. **SSB and Control Radar (SC-Radar and SC-RVM):** Uses SSB (PSS, SSS, PBCH, and PBCH DM-RS), the only always-on downlink signals, together with a PDCCH and its DM-RS, which are typically present in most 5G NR downlink configurations, to compute the CFR (2D linear interpolation).
2. **Pilot Radar (P-Radar and P-RVM):** Uses only CSI-RS signals, which are an optional part of the 5G NR downlink waveform, to compute the CFR (no interpolation required).
3. **SSB, Control and Pilot Radar (SCP-Radar and SCP-RVM):** Uses SSB, PDCCH with PDCCH DM-RS, and CSI-RS to compute the CFR (2D linear interpolation).
4. **SSB and Control, Pilot, and Data Radar (SCPD-Radar and SCPD-RVM):** Uses all downlink channels and signals (SSB, PDCCH with PDCCH DM-RS, CSI-RS, and PDSCH with PDSCH DM-RS) to compute the CFR (2D linear interpolation).
5. **SSB, Control and Data Radar (SCD-Radar and SCD-RVM):** Uses all downlink channels and signals except CSI-RS (i.e., SSB, PDCCH with PDCCH DM-RS, and PDSCH with PDSCH DM-RS) to compute the CFR (2D linear interpolation).

For clarity, these configurations are also summarized in Table 6. Since P-Radar relies exclusively on the CSI-RS, which is the only signal regularly distributed in both time and frequency, these samples were directly extracted from the OFDM grid and used for radar processing. As a result, due to regularity of CSI-RSs, no interpolation was required, but due to its sparsity, the corresponding CFR and RVM exhibited reduced resolutions. In contrast, the remaining radar configurations exploit various combinations of 5G downlink channels and signals that are irregularly distributed over the resource grid. For these cases, two-dimensional linear interpolation was applied to the estimated CFR to fill missing values prior to further radar processing.

**Figure 8:**
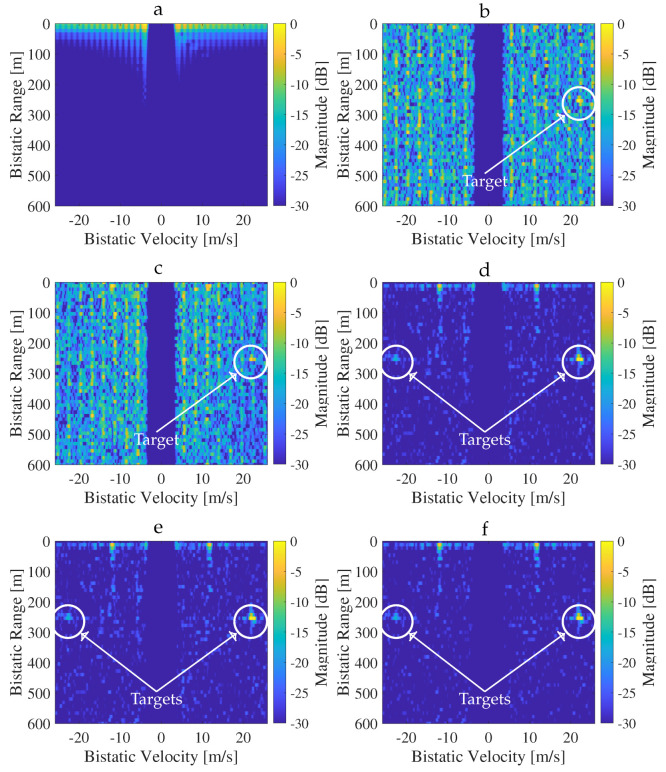
RVMs obtained from processing signal A at the 0.7 s timestamp using (**a**) SC-Radar, (**b**) P-Radar, (**c**) SCP-Radar, (**d**) SCPD-Radar, (**e**) SCD-Radar, and (**f**) GA-Radar. Clutter in the zero-Doppler region was suppressed using the ECA+ algorithm. Residual LOS clutter artifacts are visible at short ranges. Detected targets are indicated by circles.

Figure 8a–e shows the RVMs obtained from radar processing of Signal A at a timestamp of 0.7 s for each configuration. The first plot demonstrates that SC-Radar provided too few CFR samples to reliably detect targets. This issue persisted in other timestamps; therefore, in the remainder of this paper, additional results for this configuration are omitted. When using only CSI-RS (plot b) or CSI-RS combined with the SSB and PDCCH (plot c), a single target peak became visible in the RVM; however, these maps remained noticeably noisy. This situation improved significantly for the SCPD (plot d) and SCD (plot e) radars, where the addition of user data greatly increased the number of CFR samples. For these configurations, not only was the noise level reduced, but a second weaker target also became detectable in the RVM.

### 4.3. Ground-Truth Values

In the measurements, our objective was to detect vehicles moving along the high-speed road. Since the targets were non-cooperative, their exact positions and velocities were unknown. To establish a performance baseline, we applied additional processing at each timestamp using an OFDM-based radar configuration in which all RE positions and values were known (i.e., assuming a priori knowledge of the transmitted signal). Although such a scenario is unrealistic in practical bistatic systems, it provides an effective genie-aided reference for evaluating other methods. We therefore refer to this configuration as the genie-aided radar (GA-Radar). The resulting RVM is shown in Figure 8f.

After computing the RVMs, we applied a two-dimensional cell-averaging constant false alarm rate (CA-CFAR) detector [41] to identify potential targets (see Table 4). The algorithm produces a binary detection map for each RVM sample, indicating the presence or absence of the target, and it also estimates the local noise floor around each detected peak. For each timestamp, we first selected the GA-Radar CFAR detection corresponding to the highest peak energy. Since our goal was to detect vehicles within a specific region of interest, we filtered the CFAR detections using the following constraints:

1. A bistatic velocity within [−30 m/s, −10 m/s] or [10 m/s, 30 m/s].
2. A bistatic range within [180 m, 300 m].

**Figure 9:**
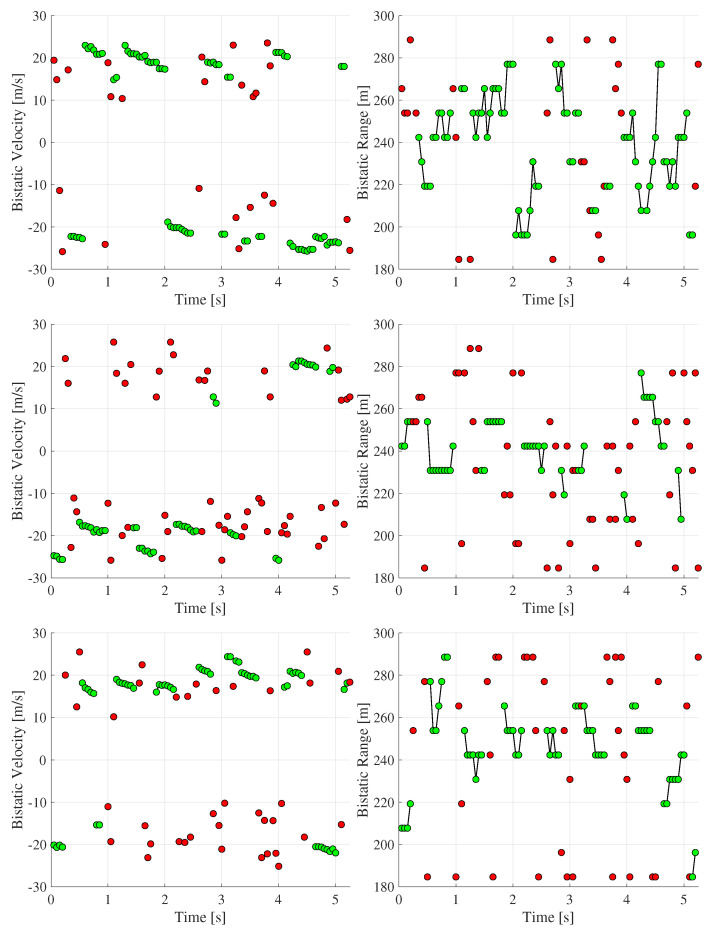
Bistatic velocity and distance of detected targets using GA-Radar for signals A (**top**), B (**middle**) and C (**bottom**). Detections associated with the same moving object are connected by straight lines. A detection is considered valid (green) only if the object is observed in at least two consecutive timestamps; otherwise, it is classified as a false detection (red).

The resulting filtered detections for each signal and each timestamp are shown in Figure 9.

To further minimize false alarms, we introduced an additional consistency check. A detection was considered valid only if the same object was observed in at least two consecutive timestamps. Two detections were treated as originating from the same object when both of the following conditions were met:

1. The difference in the bistatic velocity was below 2 m/s.
2. The difference in the bistatic range did not exceed 30 m.

In Figure 9, validated detections are shown in green, while spurious ones are marked in red. Points associated with the same moving object are linked by straight lines. As illustrated, correct detections form smooth, continuous trajectories in both velocity and range, whereas false alarms are scattered irregularly. This behavior confirms that the applied consistency rule reliably isolated the true targets.

Since no video, light detection and ranging (LiDAR) or GPS ground truth was available, the exact positions and velocities of the moving targets were unknown. In this context, the proposed GA-Radar provided the most suitable benchmark, although it has inherent limitations. In particular, it may miss targets under low SNRs or strong clutter, and its detections cannot be independently verified. Nevertheless, we believe that the filtering constraints described above effectively retain only true detections. Moreover, the estimated ranges and velocities are in line with the results reported in previous studies conducted at the same location using alternative sensing techniques, such as orthogonal time frequency space (OTFS)-based radar [42,43,44] and CAF-based radar [19,44]. We also computed CAF-based RVMs for the selected timestamps and observed moving targets at locations consistent with those detected by the GA-Radar. However, since CAF relies on correlation processing, it introduces artifacts due to repeating 5G signal components, which would require additional post-processing. Owing to these limitations and the higher computational complexity of CAF, we restricted the scope of this study only to OFDM-based radar techniques. Consequently, in the remainder of this section, only timestamps for which the GA-Radar produced valid detections (green points in Figure 9) are used to evaluate the performance of the other sensing methods, and they are called ground-truth values.

### 4.4. Radar Performance Comparison

To evaluate the performance of each sensing method, we used the PNFR, a metric representing the relative height of the detected peak. Given the CFAR-estimated noise level $A_{noise}$ and the amplitude of the detected peak $A_{echo}$, the PNFR is defined as

(6) $$\mathrm{PNFR}=20\log_{10}\left(\frac{A_{echo}}{A_{noise}}\right).$$

**Figure 10:**
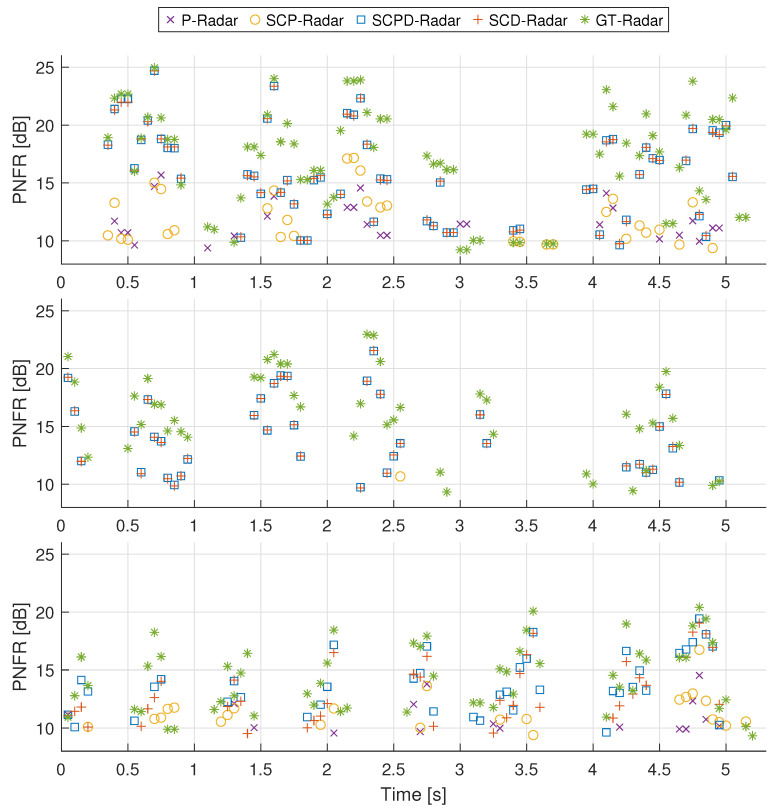
PNFR values obtained for signals A (**top**), B (**middle**) and C (**bottom**) using different radar types (see legend at the top).

For every sensing configuration, the CFAR detector was applied to the corresponding RVM at all timestamps where a ground-truth value was available. The range and velocity of all CFAR detections were then compared with the GA-Radar reference, and only those within the tolerance of 25 m and 2 m/s were preserved. From the valid detections, the result with the highest PNFR was selected. Figure 10 summarizes the PNFR values obtained through this procedure. Table 7 shows how many timestamps contained the correct target detection for each type of radar.

**Figure 11:**
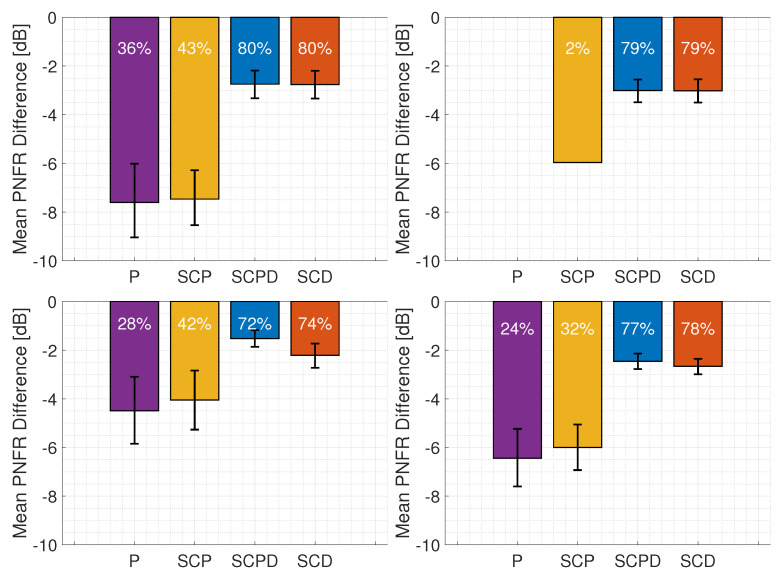
Mean (solid bars) and confidence intervals (error bars) of PNFR difference (the smaller the better) between the P-, SCP-, SCPD-, SCD-, and GA-Radars for signal A (**top-left**), signal B (**top-right**), signal C (**bottom-left**), and aggregated data from all signals (**bottom-right**). POD is displayed in white.

To better highlight the differences between the radar configurations, we also evaluated the PNFR deviation from the ground truth for each method. Figure 11 presents the mean PNFR differences for signals A, B, and C and aggregated across all datasets. We further performed a statistical analysis of the computed values. Given the limited number of valid detections in certain configurations (e.g., P-Radar in signals A and C; see Table 7), the central limit theorem (CLT) could not be invoked to assume normality. Analysis of histograms and Q-Q plots confirmed that the error distributions were non-normal; consequently, non-parametric statistical methods were employed.

First, we estimated the 95% confidence intervals for the mean PNFR differences using bootstrap resampling [45], which are shown in Figure 11. Subsequently, we conducted a Friedman test [46], a non-parametric alternative to repeated measures ANOVA, to check the null hypothesis that all radar configurations yield identical performance distributions. The test was applied to the timestamps where all radar types successfully detected the target ($N_{rows}$ in Table 8). The results, summarized in Table 8, show extremely low *p*-values (p<0.001), allowing us to confidently reject the null hypothesis. The mean ranks show two distinct performance levels, where the configurations utilizing user data (SCPD and SCD) significantly outperformed the pilot-only variants (P and SCP).

To further evaluate these differences, pairwise Wilcoxon signed-rank tests [47] were conducted. The resulting *p*-values are detailed in Table 9. Significance was evaluated against a standard threshold of α=0.05 and a Bonferroni-corrected threshold of $\alpha_{corr}=0.05/6\approx 0.0083$. For signals A and B, where the PDSCH occupied most available resources, the difference between the SCPD-Radar and SCD-Radar was not statistically significant (p>α), and their mean PNFR values were nearly identical. This indicates that adding sparse CSI-RS samples to a fully loaded PDSCH grid results in negligible gains. In contrast, for signal C, the addition of a CSI-RS led to a statistically significant improvement ($p<\alpha_{corr}$), likely because the pilots stabilized interpolation in slots lacking PDSCH data. We also observed that the PNFR values of the SCD-Radar and SCPD-Radar remained slightly below those of the GA-Radar (by approximately 2–3 dB), although they relied on a similar or even identical number of CFR samples. This degradation resulted from transmission errors in the demodulated user data, and this is further analyzed in Section 4.5.

In contrast, the radars relying primarily on pilot signals (P-Radar and SCP-Radar) showed substantially lower performance, with PNFR loss of approximately 4.5–7.5 dB and 4–7.5 dB, respectively. This confirms that the use of more samples in CFR estimation consistently improves radar performance, a conclusion supported by the pairwise comparisons in Table 9, where all pilot-based vs. data-aided comparisons resulted in *p*-values below the $\alpha_{corr}$ threshold. Finally, we highlight the detections in signal A between 3 and 4 s, where some PNFR values exceeded the ground-truth values. This behavior was likely caused by the presence of a strong peak around 60 m and 11 m/s in the RVM, which most likely originated from a slowly moving car near the receiver in the parking area. As a result, the P- and SCP-RVMs became extremely noisy in the remaining regions, which could occasionally produce spurious detections with artificially elevated PNFR values.

Similar trends appeared in the probability of detection, defined as

(7) $$POD=\frac{N_{S/C/P/D}}{N_{GA}},$$

where $N_{S/C/P/D}$ is the number of timestamps in which the radar under testing produced a detection that matched the ground-truth value within tolerances of 25 m in range and 2 m/s in velocity and $N_{GA}$ is the total number of detections reported by the GA-Radar, summarized in Table 7. The corresponding POD values are displayed on each bar in Figure 11. The SCPD-Radar and SCD-Radar detected nearly the same number of targets across all three signals. However, for signal C, their POD was noticeably lower due to the reduced amount of user data available. However, the P-Radar and SCP-Radar exhibited substantially poorer performance. For signal B, sensing with the P-Radar was entirely impossible because the CSI-RS density was too low to detect high-speed targets. Adding synchronization and control signals (SCP-Radar) offered little improvement in this case, resulting in only a single correct detection. It can also be observed that the SCP-Radar consistently detected more targets than the P-Radar in other signals.

### 4.5. BER’s Impact on Radar Performance

In a bistatic set-up, the transmitted user data contained in the PDSCH were not known at the receiver. Therefore, we demodulated the PDSCH symbols, compared the reconstructed bits with the originally generated bitstream, and computed the BER as follows:

(8) $$\mathrm{BER}=\frac{N_{\mathrm{errors}}}{N_{\mathrm{bits}}}.$$

**Figure 12:**
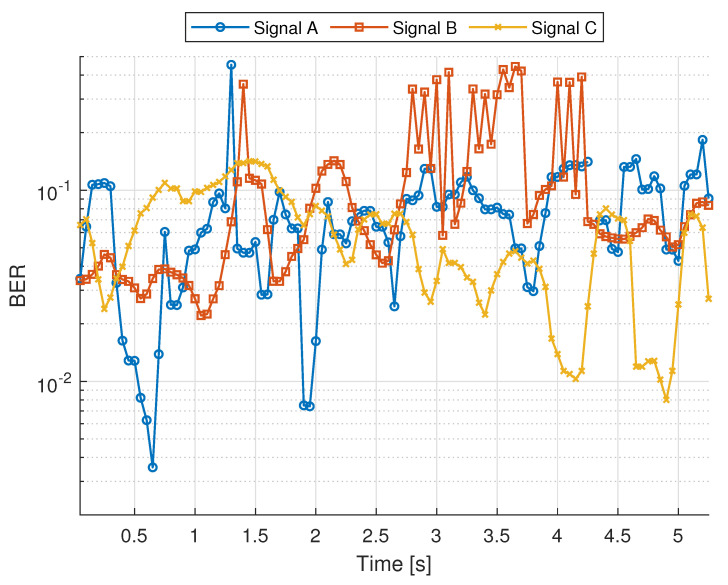
BER of user data bit (PDSCH) transmission with signals A, B, and C.

Figure 12 presents the BER obtained at each timestamp of each signal. The values ranged from 0.35% to 45%, with every signal containing segments of low (good) and high (poor) BERs. These variations arose from changes in the propagation conditions during the measurements. Since the signals were transmitted sequentially, each experienced a slightly different channel realization, leading to distinct BER fluctuations. It is also important to note that the values obtained were relatively high compared with those typically observed in real 5G deployments. This was mainly due to two factors. First, our field measurements were performed using SDR hardware, which has significantly lower RF performance than commercial 5G base stations. Second, our communication processing chain is simplified; for example, we omit forward error correction (FEC), which plays a crucial role in maintaining a low BER in practical 5G systems.

**Figure 13:**
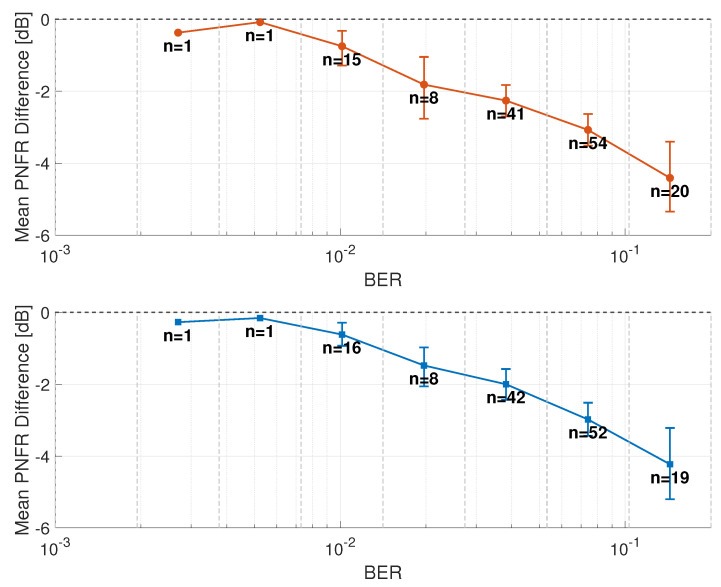
Mean PNFR difference from the ground-truth values in relation to the BER of the SCD-Radar (**top**) and SCPD-Radar (**bottom**). The number of PNFR values assigned to each BER interval is indicated by *n*. Confidence intervals are shown as error bars.

Since the sensing stage, the SCPD-Radar and SCD-Radar treated the demodulated user bits as pseudo-pilots. Without any knowledge of the actual BER, a high error rate may decrease radar performance. To evaluate this effect, we used the aggregated PNFR difference, relative to the GA-Radar, across all signals and timestamps, obtained with the SCD-Radar and SCPD-Radar. The GA-Radar represents the ideal case with a BER = 0, as it assumes perfect knowledge of the transmitted bits. The BER range was then segmented into several intervals. Each PNFR difference value was assigned to the corresponding BER interval based on the BER measured at that timestamp. For each interval, we computed the average PNFR difference. The resulting relationship between the BER and PNFR degradation is presented in Figure 13.

These results show that for low BER values, both the SCD-Radar and SCPD-Radar achieved performance only slightly worse than the GA-Radar, with differences below 0.5 dB. When the BER increased to about $10^{-2}$, the curves began to decline, and a significant performance degradation (above 4 dB) appeared once the error rate exceeded $10^{-1}$. To statistically validate this trend, we performed a Spearman rank correlation test between the BER and the PNFR degradation for both radar configurations. Crucially, this analysis was conducted on all individual BER-PNFR pairs rather than the mean values shown in Figure 13, ensuring the test captured the full variance of the dataset. The analysis showed a statistically significant positive correlation for both the SCD-Radar (ρ=0.5412, $p=5.05\times 10^{-12}$) and the SCPD-Radar ($\rho =0.5732,p=1.66\times 10^{-13}$). These results confirm with high statistical confidence that the sensing error monotonically increases as the reliability of the decoded user data decreases.

### 4.6. Radar Precision with Varying Known Data Locations

**Figure 14:**
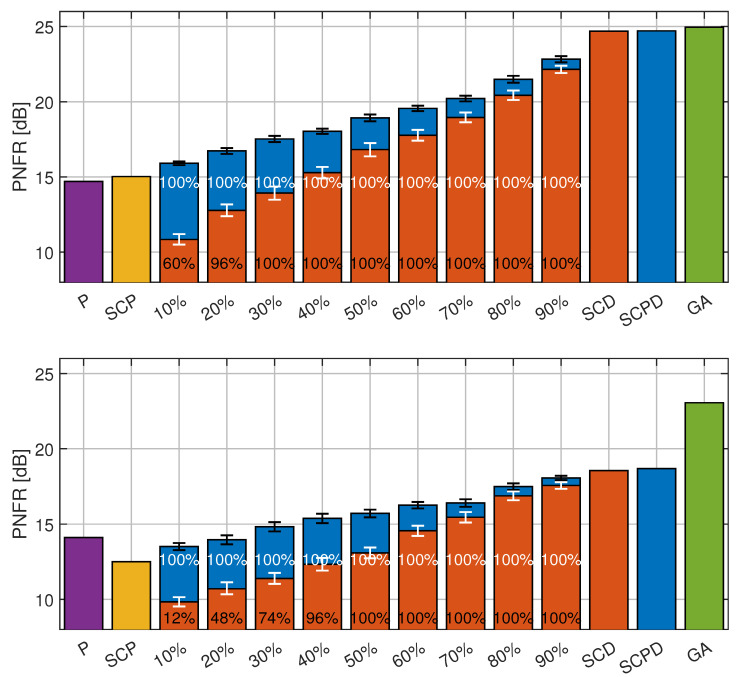
PNFR of SCD-Radar (brown) and SCPD-Radar (blue) for varying percentages of known PDSCH locations, compared against P-Radar (violet), SCP-Radar (orange), and GA-Radar (green). Results are shown for signal A at timestamp 0.7 s (**top**) and 4.1 s (**bottom**). On the color bars, the POD of the SCPD-Radar is shown in white, while the POD of the SCD-Radar is shown in black. Confidence intervals are shown as error bars.

Since having full knowledge of all PDSCH allocations and their DM-RS is unrealistic in a real 5G deployment, we investigated scenarios where only a portion of the PDSCH positions was known to the ISAC receiver, similar to the case where the receiver is connected to the network and decodes only assigned or shared resources. To emulate this, the received 5G OFDM grid (1596 × 2800 RE) was partitioned into 104 equally sized fragments (200 × 215). A subset of these fragments was then randomly selected, and only the PDSCH resources within these fragments were demodulated, reconstructed, and used, together with their DM-RS, for radar sensing. Figure 14 shows the results of this experiment for signal A at timestamp 0.7 s, where the BER was relatively low (about 0.014), and at timestamp 4.1 s, where the BER was high (about 0.135). For both the SCPD-Radar and SCD-Radar, we randomly selected a given percentage of fragments (10%, …, 90%) 50 times, performed sensing, and compared the output with the ground-truth values. For each bar, the POD is printed in white for the SCPD-Radar and in black for the SCD-Radar, while the bar height corresponds to the mean PNFR of runs with successful detection.

The results for both timestamps show that adding any amount of PDSCH data consistently increased the PNFR for both the SCD-Radar and SCPD-Radar. The only exception appeared at timestamp 4.1, when only a small number of PDSCH samples was available (SCP = 0%, 10%, 20%). In this situation, the low number of additional and highly erroneous data slightly degraded the PNFR of the SCPD-Radar against the P-Radar, likely due to interpolation inaccuracies. To statistically verify the impact of data availability on sensing accuracy, we computed the Spearman rank correlation using the complete set of individual measurement pairs, correlating the percentage of available PDSCH REs directly with the resulting PNFR values (percent of known data locations vs. PNFR). The analysis confirmed a strong, statistically significant positive correlation for both radar types, with *p*-values approaching zero for all cases. At timestamp 0.7, we observed ρ=0.95 for the SCPD-Radar and ρ=0.93 for the SCD-Radar. Similarly, at timestamp 4.1, the correlation remained strong, with ρ=0.84 for the SCPD-Radar and ρ=0.89 for the SCD-Radar. A comparison of POD values using Fisher’s exact test [48] revealed distinct data requirements for the two configurations. In the low-BER conditions (timestamp 0.7), the SCD-Radar required only 20% of the user data to effectively match the SCPD-Radar detection rate; at 10%, the difference remained significant (p<0.0001), but at 20%, it became statistically negligible (p=0.25). In contrast, the high-BER conditions (timestamp 4.1) demanded a higher data threshold. Statistically significant differences persisted at 10%, 20%, and 30% data availability (p<0.0001), with detection performance becoming indistinguishable only when 40% of the PDSCH REs were utilized (p=0.25).

## 5. Radar Sensitivity to User Data QAM Order

**Figure 15:**
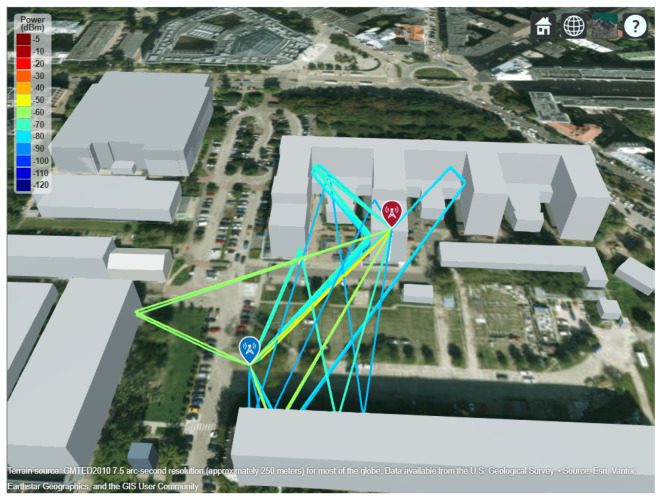
Simulation geometry. Screenshot from the MATLAB site viewer showing OpenStreetMap buildings (gray blocks) after placing the transmitter (red indicator) and receiver (blue indicator) and performing ray-tracing simulations (lines).

All signals transmitted during our field measurements used 4-QAM modulation for the PDSCH. However, real 5G deployments use adaptive QAM orders, depending on the modulation and coding scheme (MCS), which is dynamically adjusted based on channel quality to keep the BER low. Higher-order QAM constellation states have different amplitudes, making them more sensitive to noise and potentially affecting radar performance. To complement our field results, we therefore show the initial results from the simulations to assess how an increased QAM order influences sensing accuracy. To keep the simulation environment realistic, we exported the measurement location from OpenStreetMap and imported it into MATLAB. The transmitter, receiver, and carrier parameters were configured to match the field set-up. Then, ray tracing was performed using the built-in functions of MATLAB [49], producing propagation paths between the transmitter and receiver along with their characteristics (path loss, phase shift, and propagation delay). The simulation geometry and the extracted paths are shown in Figure 15.

**Figure 16:**
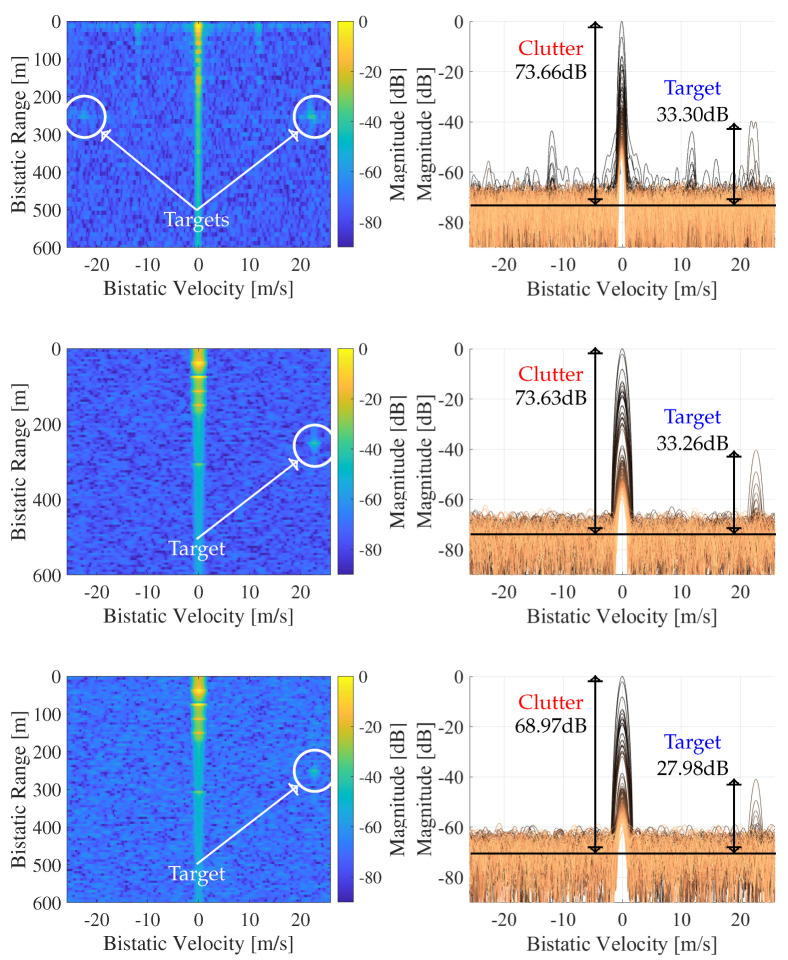
RVMs (**left**) and Doppler profiles (**right**) after processing (without ECA+) of signal A: field measurements with 4-QAM (**top**); simulations with 4-QAM (**middle**); and simulations with 256-QAM (**bottom**). Targets are indicated by circles.

Because the exported geometry did not include moving vehicles, we manually inserted an additional dynamic path representing a moving object. Its parameters, namely delay, Doppler, and complex gain, were taken from the strongest target peak observed in the RVM of Signal A at timestamp 0.7 s (Figure 8). Using the complete set of paths, we generated the received signal according to Equation (1). We simulated Signal A using both 4-QAM and 256-QAM and added AWGN, matching the noise level measured in the field. Finally, radar processing was performed using the GA-Radar. Figure 16 presents the resulting radar output.

The results indicate that the difference in the PNFR between using 4-QAM and 256-QAM was approximately 4.5 dB. It is important to note that these results were obtained using the GA-Radar, where all transmitted user bits were known a priori. Under the high-noise conditions in this simulation, the SCPD-Radar and SCD-Radar could not operate with 256-QAM because the transmission BER was extremely high (about 38%), making symbol reconstruction impossible. However, real 5G systems are designed to avoid such situations; the MCS is continuously adapted to maintain a low BER, meaning that 256-QAM would never be selected under these poor channel conditions. In summary, our initial investigation suggests that when the channel quality degrades, the 5G network will reduce the QAM order, which in turn improves radar performance. In contrast, when the channel quality is good, higher QAM orders are used, which leads to slightly worse radar performance compared with the 4-QAM case. On the other hand, in those conditions, the noise floor is also lower, enabling the radar to operate reliably despite the higher QAM order. Still, these conclusions are based on preliminary simulations and should be validated in future work through more extensive simulations and field measurements.

## 6. Conclusions

In this paper, we experimentally evaluated 5G OFDM-based radar systems that jointly exploit control, reference, and user data resources. Our findings show that incorporating transmitted user data, even when reconstructed with errors, significantly improves sensing performance compared with pilot-only approaches. First, we compared the PNFR and POD across radars using different combinations of 5G downlink waveform components. The results indicate that the POD was relatively low for the CSI-RS-only radar (P-Radar), reaching only 24% on average. Adding an SSB and PDCCH (SCP-Radar) increased the POD to 32%. The best performance was achieved when user data was included; the POD reached 77% for the SCPD-Radar and 78% for the SCD-Radar. The PNFR followed the same trend; radars using PDSCHs achieved values only about 2.5 dB below the ground-truth reference, whereas the pilot-only radars exhibited a much larger deviation of roughly 6.5 dB. Next, we analyzed how transmission errors affect radars that utilize PDSCHs (SCD-Radar and SCPD-Radar). For a low BER (below $10^{-2}$), the mean difference in the PNFR from the value of the ground truth remained small (around 0.5 dB). As the BER increased, PNFR degradation became noticeable, reaching approximately 4.5–5 dB for BERs above $10^{-1}$. Finally, we investigated how the number of known PDSCH locations influenced the quality of the detections. When the target was detectable even with a CSI-RS alone (P-Radar), any additional user data improved the PNFR, with the POD remaining at 100%. However, when a CSI-RS was not available, achieving a POD greater than 96% required knowledge of at least 20% of the PDSCH REs under low-BER conditions and 40% under high-BER conditions. In these scenarios, increasing the number of known PDSCH positions also increased the PNFR. Furthermore, we acknowledge that the reported PNFR and POD values were obtained under specific channel conditions and with custom hardware and software, which may differ from those in operational 5G networks. As a result, the absolute PNFR and POD values may vary under different environments or system configurations. Nevertheless, we expect that the overall trends observed in this work will remain valid across a wide range of deployment scenarios.

In addition to the experimental verification, we also presented preliminary simulation results that illustrate how different QAM orders may affect the detection performance. Under conditions similar to those in our field measurements, we observed that the PNFR difference between 4-QAM and 256-QAM, when sensing with the GA-Radar (assuming full a priori knowledge of PDSCH symbols), was approximately 4.5 dB. We also note that 5G networks dynamically adapt the QAM order based on the channel quality to maintain a low BER. This mechanism can be beneficial for sensing; in difficult conditions, the system switches to lower-order modulation, which supports better sensing performance, while in good conditions, the noise floor is already low, and the higher QAM order results only in a small PNFR reduction. Nevertheless, this statement is based only on simplified simulations; therefore, we consider the impact of adaptive modulation to be an important direction for future work. It is also worth noting that our communication processing pipeline is simplified and does not include FEC, resulting in a higher BER than in real 5G systems. Therefore, as part of future developments, we aim to improve our sensing set-up and perform measurements using real (non-SDR) 4G and 5G base stations. This would ultimately validate that bistatic radar systems can be implemented on existing network infrastructure without requiring major modifications.

## Figures and Tables

**Table 1:** 5G NR parameters of test signals A, B, and C.

Parameter	Value	Comment
$N_{\mathrm{fft}}$	2048	FFT length
$N_{\mathrm{used}}$	1596	Number of used carriers
$N_{\mathrm{cp}1},N_{\mathrm{cp}2}$	144, 176	Length of cyclic prefixes
$f_{c}$	5.8 [GHz]	Carrier frequency
$f_{s}$	61.44 [MHz]	Sampling frequency
SCS	30 [kHz]	Subcarrier spacing
ssburst.Period	20 [ms]	Allocation period
ssburst.TransmittedBlocks	[0 1 0 1]	Active SSBs in SS burst
pdcch.SlotAllocation	0	Allocated slot indices
pdcch.Period	5 [slots]	Allocation period
pdsch.Modulation	‘QPSK’ (4-QAM)	User data modulation type
pdsch.SymbolAllocation	[0 14] (A, B); [2 10] (C)	First symbol and length
pdsch.SlotAllocation	[0:19] (A, B); [3:39] (C)	Allocated slot indices
pdsch.Period	20 (A, B); 40 (C) [slots]	Allocation period
pdsch.DMRS.SubcarrierLocations	[0, 2, 4, 6, 8, 10]	Freq. locations (per 12 REs)
pdsch.DMRS.AdditionalPosition	0	Only 1 symbol per slot
pdsch.DMRS.TypeAPosition	2 [symbol]	Location in slot
csirs.Density	“one”	Frequency density (1/12 REs)
csirs.SubcarrierLocations	6 [subcarrier]	Freq. locations (per 12 REs)
csirs.SymbolLocations	13 [symbol]	Symbol locations in slot
csirs.CSIRSPeriod	1 (A, C); 4 (B)	Allocation period in slots

**Table 2:** Parameters of the SDR and PC platform.

Parameter	Specification
SDR type	NI USRP 2953R (USRP X310)
SDR daughterboards	UBX-160
SDR synchronization	GPS disciplined oscillator
SDR interface	PCI-Express x4
SDR sampling frequency	33.33 [MHz]
Beamwidth	16°
Directivity	19 dBi
Amplifier gain	20 dB

**Table 3:** Parameters of the ECA+ algorithm [38,39].

Parameter	Value
Doppler removal area	−3.14:3.14 [m/s]
Delay removal range	0:1662 [m]

**Table 4:** Parameters of the CFAR algorithm [41].

Parameter	Value
Training band size	[4 6]
Guard band size	[2 8]
Probability false alarm	$1\times 10^{-3}$
Method	CA (cell-averaging)

**Table 5:** Comparison of radar metrics between the nominal 5G NR sampling rate (61.44 MHz) and the hardware-limited SDR sampling rate (33.33 MHz).

Parameter	5G NR fs = 61.44 MHz	SDR fs = 33.33 MHz
Range resolution [m]	6.26	11.54
Velocity resolution [m/s]	0.052	0.028
Max velocity [m/s]	723	392

**Table 6:** Specification of tested 5G OFDM-based radar combinations.

Radar Denotation	SSB	PDCCH and PDCCH DM-RS	PDSCH and PDSCH DM-RS	CSI-RS	2D Interpolation
SC-Radar	Yes	Yes	No	No	Yes
P-Radar	No	No	No	Yes	No
SCP-Radar	Yes	Yes	No	Yes	Yes
SCPD-Radar	Yes	Yes	Yes	Yes	Yes
SCD-Radar	Yes	Yes	Yes	No	Yes

**Table 7:** Number of correct object (vehicle) detections for different radar types.

Signal	$N_{P}$	$N_{\mathrm{SCP}}$	$N_{\mathrm{SCPD}}$	$N_{\mathrm{SCD}}$	$N_{\mathrm{GA}}$
A	27	32	60	60	75
B	0	1	38	38	48
C	16	24	41	42	57
All	43	57	139	140	180

**Table 8:** Friedman test results and mean ranks for mean PNFR difference between different radar configurations and GA-Radar (excluding signal B due to $N_{P}=0$ and $N_{SCP}=1$).

Signal	$N_{\mathrm{rows}}$	*p*-value	$R_{P}$	$R_{\mathrm{SCP}}$	$R_{\mathrm{SCPD}}$	$R_{\mathrm{SCD}}$
A	18	$6\times 10^{-10}$	3.72	3.28	1.22	1.78
C	11	$1\times 10^{-5}$	3.82	3.09	1.45	1.64
Both	29	$2\times 10^{-15}$	3.76	3.21	1.31	1.72

**Table 9:** The *p*-values of the pairwise Wilcoxon signed-rank tests for the mean PNFR difference between the radar configurations and GA-Radar. For signal B, some values are not reported due to insufficient sample counts ($N_{P}=0$ and $N_{SCP}=1$), and are indicated by “—”.

Signal	vs. P-Radar			vs. SCP-Radar		Data-Only
	SCP	SCPD	SCD	SCPD	SCD	SCPD vs. SCD
A	0.0766	<0.0001	<0.0001	<0.0001	<0.0001	0.9121
B	—	—	—	—	—	0.5281
C	0.0098	0.0006	0.0006	0.0002	0.0002	0.0068
All	0.0036	<0.0001	<0.0001	<0.0001	<0.0001	0.0396

## Data Availability

The original contributions presented in the study are included in the article. Further inquiries can be directed to the corresponding author.

## Abbreviations

The following abbreviations are used in this manuscript:

BER	Bit error rate
C(-Radar/RVM)	Control (Radar-RVM)
CA-CFAR	Cell-Averaging Constant False Alarm Rate
CAF	Cross-ambiguity function
CFO	Carrier frequency offset
CSI-RS	Channel state information reference signal
CP	Cyclic crefix
D(-Radar/RVM)	Data (Radar/RVM)
DDM/DDR	Delay-Doppler map/delay-Doppler response
DM-RS	Demodulation reference signal
DV-TR	Doppler-variant transfer response
ECA	Extensive Cancellation Algorithm
GT(-Radar/RVM)	Ground truth (Radar/RVM)
(I)FFT	(Inverse) Fast Fourier transform
ISAC	Integrated sensing and communication
ICI	Intercarrier interference
ISI	Intersymbol interference
LOS	Line-of-sight
LTE	Long-term evolution
LTI	Linear time-invariant
LTV	Linear time-variant
OFDM	Orthogonal frequency division multiplexing
NR	New Radio
P(-Radar/RVM)	Pilot (Radar/RVM)
PBCH	Physical broadcast channel
PDCCH	Physical downlink control channel
PDSCH	Physical downlink shared channel
PNFR	Peak-to-noise-floor ratio
POD	Probability of detection
PRS	Positioning reference signal
PSS	Primary synchronization signal
PT-RS	Phase tracking reference signal
QAM	Quadrature amplitude modulation
RE	Resource element
RMSE	Root mean square error

RNTI	Radio Network Temporary Identifier
RVM	Range–velocity map
S(-Radar/RVM)	Synchronization/SSB (Radar/RVM)
SCS	Subcarrier spacing
SDR	Software-Defined Radio
SSB	Synchronization signal block (SSB)
SSS	Secondary synchronization signal
TRS	Tracking reference signal
(TV-)CFR	(Time-variant) channel frequency response
(TV-)CIR	(Time-variant) channel impulse response
UD(-Radar/RVM)	User data (Radar/RVM)
